# Beginning Restorative Activities Very Early: Implementation of an Early Mobility Initiative in a Pediatric Onco-Critical Care Unit

**DOI:** 10.3389/fonc.2021.645716

**Published:** 2021-03-08

**Authors:** Saad Ghafoor, Kimberly Fan, Sarah Williams, Amanda Brown, Sarah Bowman, Kenneth L. Pettit, Shilpa Gorantla, Rebecca Quillivan, Sarah Schwartzberg, Amanda Curry, Lucy Parkhurst, Marshay James, Jennifer Smith, Kristin Canavera, Andrew Elliott, Michael Frett, Deni Trone, Jacqueline Butrum-Sullivan, Cynthia Barger, Mary Lorino, Jennifer Mazur, Mandi Dodson, Morgan Melancon, Leigh Anne Hall, Jason Rains, Yvonne Avent, Jonathan Burlison, Fang Wang, Haitao Pan, Mary Anne Lenk, R. Ray Morrison, Sapna R. Kudchadkar

**Affiliations:** ^1^ Division of Critical Care Medicine, Department of Pediatric Medicine, St. Jude Children’s Research Hospital, Memphis, TN, United States; ^2^ Department of Pediatric Critical Care, University of Tennessee Health Science Center, Memphis, TN, United States; ^3^ Office of Quality and Patient Care, St. Jude Children’s Research Hospital, Memphis, TN, United States; ^4^ Department of Rehabilitation Services, St. Jude Children’s Research Hospital, Memphis, TN, United States; ^5^ Department of Child Life, St. Jude Children’s Research Hospital, Memphis, TN, United States; ^6^ Department of Psychology, St. Jude Children’s Research Hospital, Memphis, TN, United States; ^7^ Division of Psychiatry, Department of Pediatric Medicine, St. Jude Children’s Research Hospital, Memphis, TN, United States; ^8^ Division of Anesthesiology, Department of Pediatric Medicine, St. Jude Children’s Research Hospital, Memphis, TN, United States; ^9^ Department of Pharmaceutical Services, St. Jude Children’s Research Hospital, Memphis, TN, United States; ^10^ Department Critical Care/Pulmonary Medicine-Respiratory Therapy, St. Jude Children’s Research Hospital, Memphis, TN, United States; ^11^ Department of Inpatient Units-Nursing, St. Jude Children’s Research Hospital, Memphis, TN, United States; ^12^ Department of Nursing Administration- Nursing Education, St. Jude Children’s Research Hospital, Memphis, TN, United States; ^13^ Department of Pharmaceutical Sciences- Patient Safety, St. Jude Children’s Research Hospital, Memphis, TN, United States; ^14^ Department of Biostatistics, St. Jude Children’s Research Hospital, Memphis, TN, United States; ^15^ Department of Quality Improvement Education and Training, Cincinnati Children’s Hospital- James M. Anderson Center for Health Systems Excellence, Cincinnati, OH, United States; ^16^ Departments of Anesthesiology and Critical Care Medicine, Pediatrics and Physical Medicine and Rehabilitation, Johns Hopkins University School of Medicine, Baltimore, MD, United States

**Keywords:** post-intensive care syndrome, pediatric oncology, early mobility, physical therapy, occupational therapy, delirium, quality improvement

## Abstract

**Introduction:**

Children with underlying oncologic and hematologic diseases who require critical care services have unique risk factors for developing functional impairments from pediatric post-intensive care syndrome (PICS-p). Early mobilization and rehabilitation programs offer a promising approach for mitigating the effects of PICS-p in oncology patients but have not yet been studied in this high-risk population.

**Methods:**

We describe the development and feasibility of implementing an early mobility quality improvement initiative in a dedicated pediatric onco-critical care unit. Our primary outcomes include the percentage of patients with consults for rehabilitation services within 72 h of admission, the percentage of patients who are mobilized within 72 h of admission, and the percentage of patients with a positive delirium screen after 48 h of admission.

**Results:**

Between January 2019 and June 2020, we significantly increased the proportion of patients with consults ordered for rehabilitation services within 72 h of admission from 25 to 56% (*p*<0.001), increased the percentage of patients who were mobilized within 72 h of admission to the intensive care unit from 21 to 30% (*p*=0.02), and observed a decrease in patients with positive delirium screens from 43 to 37% (*p*=0.46). The early mobility initiative was not associated with an increase in unplanned extubations, unintentional removal of central venous catheters, or injury to patient or staff.

**Conclusions:**

Our experience supports the safety and feasibility of early mobility initiatives in pediatric onco-critical care. Additional evaluation is needed to determine the effects of early mobilization on patient outcomes.

## Introduction

Survivorship for children with malignancies has significantly improved in recent decades as a result of improved understanding of cancer genomics and immunology, diagnostic modalities, risk stratification, targeted therapies, and early recognition and treatment of complications (1–5). However, up to 40% of these children still require critical care therapies for factors specific to their oncologic disease (6). These factors include immunosuppression and a dysregulated inflammatory response secondary to malignant bone marrow infiltration and chronic glucocorticoid use, infection from long-term indwelling central venous catheters, and acute and chronic organ toxicity from chemotherapy agents (7). Additionally, patients who undergo hematopoietic cell transplant (HCT) are subject to unique complications such as graft-versus-host disease, idiopathic pneumonia syndrome, diffuse alveolar hemorrhage, and sinusoidal obstruction syndrome, which further increases critical care utilization, intensive care unit (ICU) morbidity, and mortality in these patients (7–12). Furthermore, post-HCT patients and those with underlying malignancies who require critical care have higher rates of resource utilization, such as invasive mechanical ventilation, vasoactive infusions, and continuous renal replacement therapy, as well as higher mortality, when compared to the general inpatient pediatric population (6).

In recent years, with an overall improvement in ICU mortality, there has been a paradigm shift towards decreasing patient morbidity both during hospitalization and after discharge (13). However, owing to the pathobiology common to critically ill patients, such as those with sepsis, respiratory failure, cardiovascular collapse, and trauma, the focus of critical care treatment has historically been centered around establishing and maintaining hemodynamic stability, endotracheal intubation, and mechanical ventilation, which commonly involves immobilization and sedation. This kind of care often requires the use of opiate analgesics and benzodiazepines, produces continuous noise in the critical care environment, and entails frequent nursing care and invasive interventions (14–16). Although these interventions may be necessary, they disrupt the sleep-wake cycle, increase delirium, impair immunity, cause catabolism, and lead to other chronic physiologic impairments such as disuse atrophy of lean muscle mass, pressure ulcer formation, worsened pulmonary function and cardiac indices, and insulin resistance (15, 17). These sequelae put patients at risk of pediatric post-intensive care syndrome (PICS-p), a constellation of physical, cognitive, emotional, and social impairments seen in children and their caregivers, even after hospital discharge (14, 18–23). Additionally, an underlying oncologic diagnosis has been identified as an independent risk factor for acquiring critical care-related functional and cognitive impairments in pediatric patients (24).

Early mobility is a promising therapeutic option that addresses many of these issues. Although adult studies suggest clinical benefit from early mobility and rehabilitation (25–27), the use of rehabilitation resources is low in the pediatric population, with a point prevalence of 35–39% (28, 29). Early mobility-based rehabilitation programs designed to increase mobilization within 72 h of ICU admission in pediatrics are reported to be both safe and feasible and to increase physical/occupational therapy consults and early mobilization events (30–34). However, given the unique needs of the pediatric oncologic population, which has high rates of critical illness, acuity, and mortality, we identified a need to develop and implement an early mobility-based rehabilitation program (BRAVE—Beginning Restorative Activities Very Early) in our onco-critical care unit and evaluate safety and feasibility.

## Methods

### Overview of Project Design and Setting

The BRAVE early mobility initiative is a quality improvement (QI) project that was developed with the global aim of improving short- and long-term functional outcomes by decreasing the prevalence and effects of PICS-p in an onco-critical care unit. BRAVE was designed as a multidisciplinary, collaborative approach to change culture and practice through integration of the 4 Es: engage, educate, execute, and evaluate (35). BRAVE was adapted from the Johns Hopkins PICU Up! early mobility program (30) to meet the needs of our specialty unit.

BRAVE was implemented in the ICU of St. Jude Children’s Research Hospital (St. Jude), an academic, quaternary care center focused on providing medical care for children with a wide range of oncologic and hematologic disorders. The ICU is a combined medical–surgical unit consisting of eight critical care beds and four step-down beds, all single-patient rooms with an attached parent room. This ICU provides care for children with underlying malignancies, hematologic disorders, and those who have undergone HCT. Children ages 1 day to 21 years who required ICU or step-down admission were eligible for early mobility. Exclusion criteria included patients with an open chest or abdomen, unstable fractures, or with provider-placed medical order specifying otherwise. The BRAVE initiative was implemented without any additional personnel or equipment resources.

### Data Acquisition

A retrospective review of the medical records for all critical care and step-down admissions from January 2019 through June 2020 was performed. The analysis was divided into two 9-month periods: pre-BRAVE implementation (January-September 2019) and post-BRAVE implementation (October 2019–June 2020). This QI project was comprehensively reviewed and acknowledged as “Non-Human Subjects Research- Quality Improvement” by the St. Jude Institutional Review Board. Demographics, mobility data, and delirium screens were obtained from the electronic medical records. Illness severity index was provided by Virtual Pediatric Systems, LLC.

### Quality Improvement Process

For this QI initiative, we created an interprofessional team called the BRAVE Core Group. Participation in the BRAVE Core Group was open to all interested staff and had representative champions from each of the following professions: critical care physicians, advanced practitioners (AP), nursing staff, occupational therapy (OT), physical therapy (PT), child life, speech language pathology, respiratory therapy, psychology, psychiatry, pain team, and rehabilitation medicine, quality/patient care, and clinical analytics. This group of early mobility champions met weekly for over 12 months to engage and educate ICU staff on this QI project prior to its execution and evaluation.

#### Engagement

Because of the interprofessional collaboration required for a successful early mobility initiative, members from each of the disciplinary teams within the BRAVE Core Group conducted focus groups to discuss the problem, identify potential facilitators, and address potential barriers to early mobility. Based on feedback from these focus groups and review of the available medical literature, the B.R.A.V.E Core Group developed specific guidelines to safely and effectively implement early mobility in the pediatric ICU. These guidelines outline the different activity levels, required resources, and criteria for cessation and reevaluation of an activity. Leaders from the Johns Hopkins PICU Up! Program presented a hospital-wide Grand Rounds to generate institution-wide enthusiasm for this early mobility initiative.

#### Education

Educational resources regarding the BRAVE initiative were developed for all staff members who treat patients in the ICU. In October 2018, members of the BRAVE Core Group attended the Johns Hopkins Critical Care Rehabilitation Conference to learn from the experiences of the PICU-Up! early mobility team (30). Using information obtained from this conference, the BRAVE Core Group developed formal education materials including a required online learning module for all ICU teams and rotating specialty teams, simulation training for nursing staff and respiratory therapists, and educational handouts for families on admission to the critical care unit (**Supplementary Figure 1**). The online module provided a review of the early mobility literature in critically ill patients, an overview of the BRAVE initiative, and several interactive case-based scenarios designed to illustrate the application of BRAVE. Additional educational materials included lectures by the team leader on early mobility, delirium, and ventilator asynchrony, as well as handouts and pocket cards that summarized the levels and activities of BRAVE early mobility.

#### Execution

**Figure 1:**
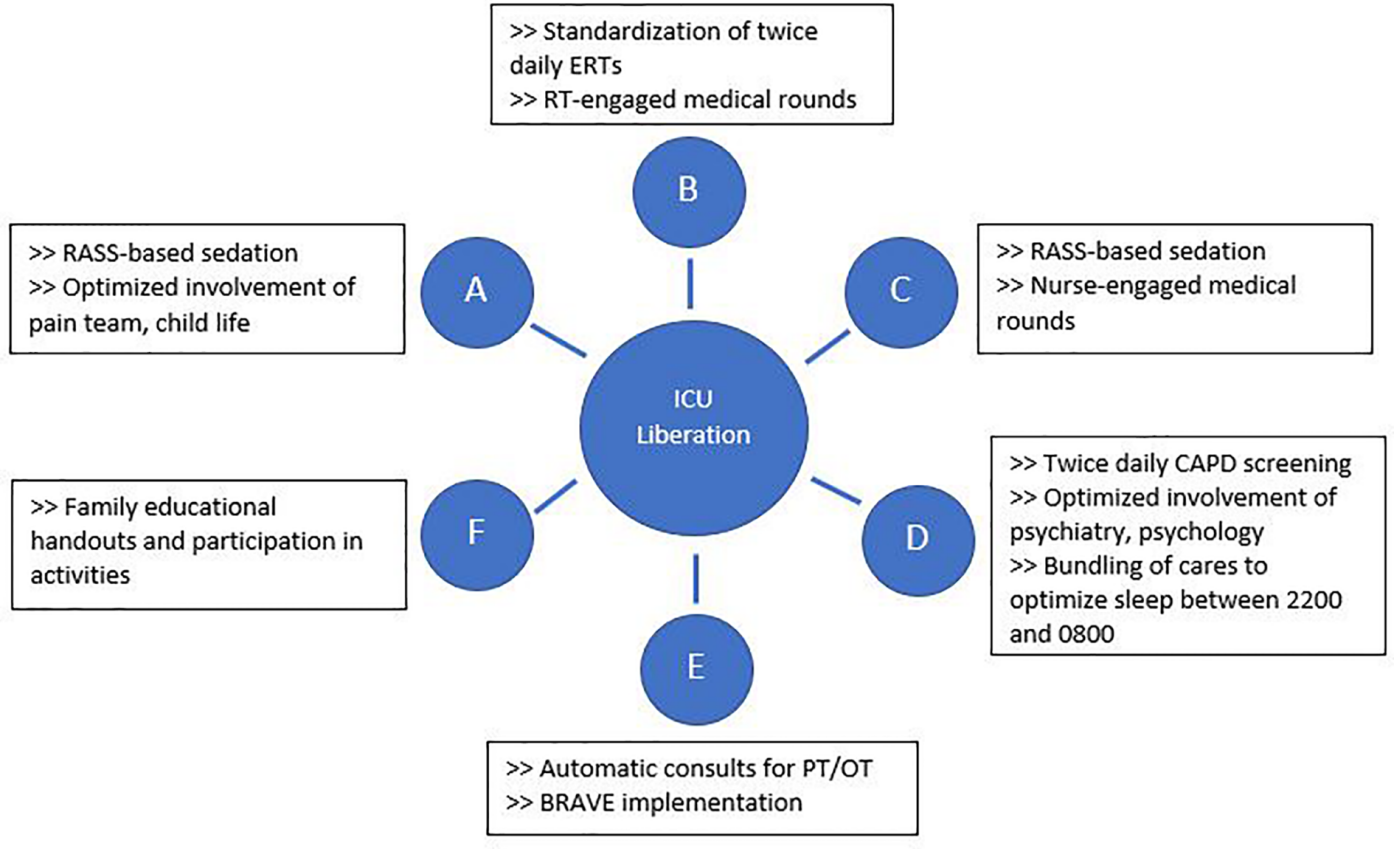
Interventions implemented through Plan-Do-Study-Act cycles to address each aspect of the ABCDEF intensive care unit liberation bundle. BRAVE, Beginning Restorative Activities Very Early; CAPD, Cornell Assessment of Pediatric Delirium; ERTs, extubation readiness trials; OT, occupational therapy; PT, physical therapy; RT, respiratory therapists.

The BRAVE Core Group used the ABCDEF ICU liberation bundle as a template to develop SMART aims and key drivers targeting early mobilization and delirium (36–39). Although early mobility and delirium represent only two components of the ABCDEF bundle, the other aspects must also be addressed to make early mobility possible and to decrease the effects of PICS-p in the long term. As such, BRAVE incorporates early mobility within a broader context of pain/delirium management, optimizing extubations, family/caregiver involvement, and good sleep. As part of BRAVE, we implemented serial Plan-Do-Study-Act (PDSA) cycles to target each component of the ABCDEF bundle (39) (**Figure 1**).

**Figure 2:**
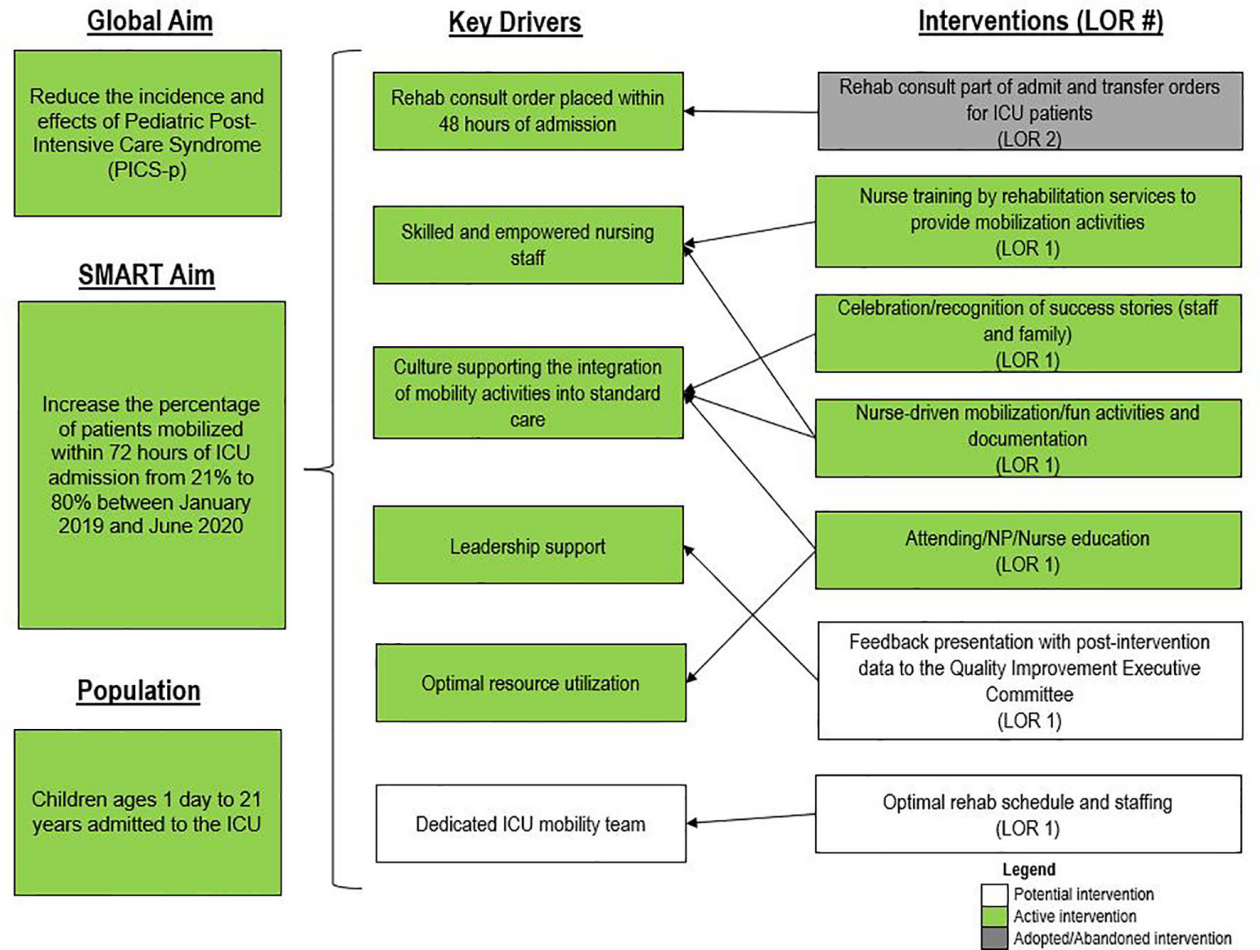
Key driver diagram targeting early mobility of intensive care unit (ICU) patients. LOR, level of reliability; NP, nurse practitioner.

**Figure 3:**
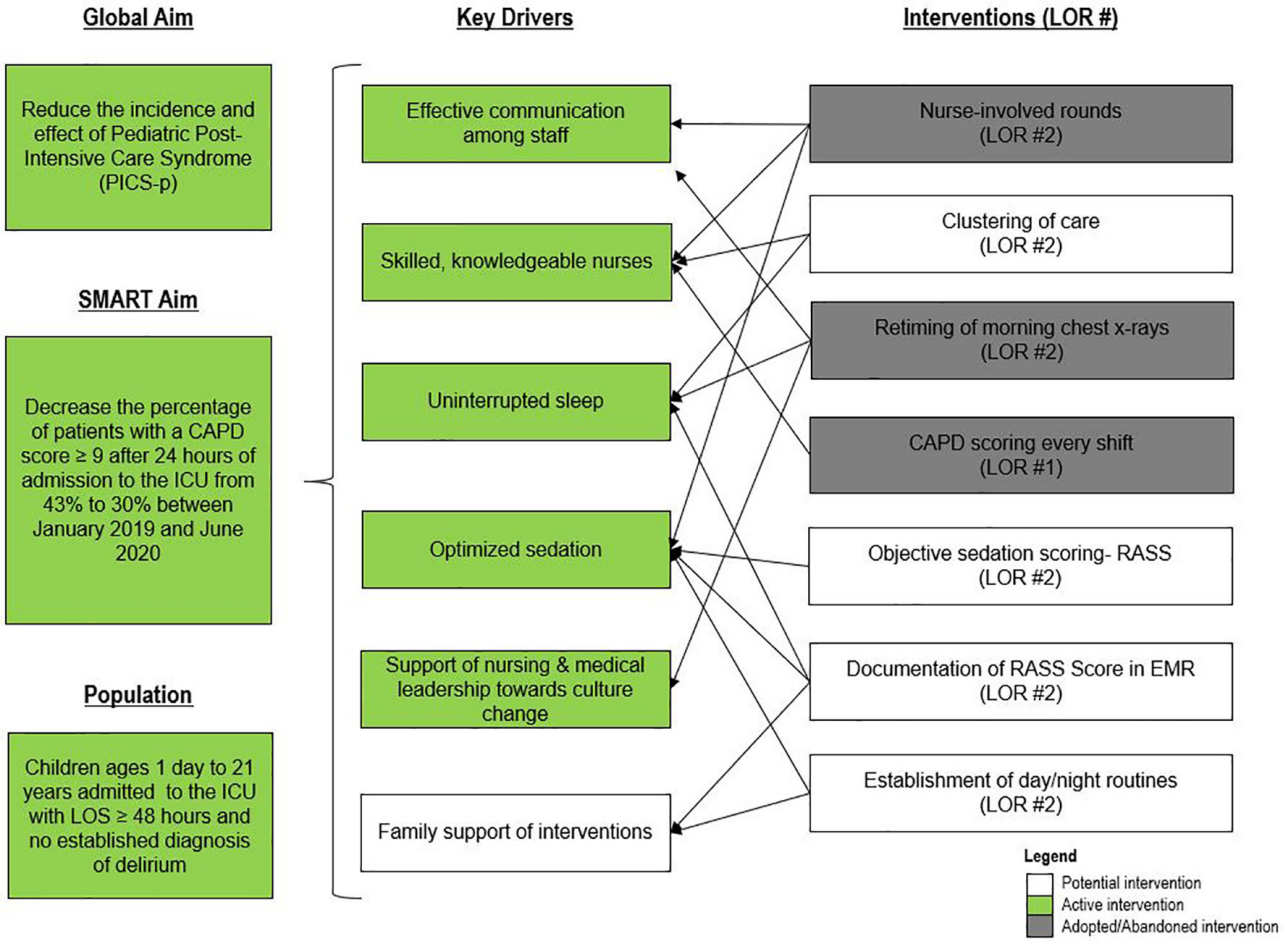
Key driver diagram targeting delirium. CAPD, Cornell Assessment of Pediatric Delirium; ICU, intensive care unit; LOR, level of reliability; LOS, length of stay; RASS, Richmond Agitation Sedation Scale.

For early mobility, the SMART aim was to increase the percent of patients mobilized within 72 h of admission from our baseline of 21% to 80% within 9 months of implementation (**Figure 2**). For delirium, the SMART aim was to decrease the proportion of pediatric ICU patients with a positive delirium screen after 24 h of admission from 43% to 30% within 9 months of implementation (**Figure 3**). Specific key drivers included empowering skilled, knowledgeable nurses to integrate mobilization activities early in care, encouraging effective communication among pediatric ICU staff, optimizing order entry for rehabilitation service consultations, targeting appropriate sedation for safe activity participation, standardizing extubation readiness trials, and allowing for uninterrupted sleep at night. These key drivers were implemented through various PDSA cycles, such as developing a delirium pathway that includes standardized delirium screening with the Cornell Assessment for Pediatric Delirium (CAPD) (40, 41), nurse-engaged rounds, streamlining nursing documentation of sedation and delirium scores, making extubation readiness trail discussions a daily part of rounds, and integrating PT and OT consults as part of the ICU admission order set.

#### Evaluation and Outcome Measures

After the start of BRAVE, the Core Group met bimonthly to review outcomes, evaluate the effects of each PDSA cycle, and discuss changes as necessary. Additionally, the group sent an anonymous response questionnaire to the entire pediatric ICU staff to collect general feedback about early mobility and ongoing barriers to mobilizing the critically ill child. At the conclusion of the post- implementation period, the group reviewed the data to evaluate the primary and secondary outcomes using a pre-/post- design.

In keeping with our SMART aims, the primary outcome measures for this study were the proportion of patients with physician- or AP- placed consult orders for OT and/or PT within 72 h of ICU admission, the proportion of patients who received an early mobility activity provided by rehabilitative services, and the percentage of positive delirium screens after 24 h of ICU admission. For early mobility, we evaluated outcomes in two cohorts of patients, those with length of stay (LOS) > 48 h and those with all LOS. For delirium screens, we evaluated patients with LOS > 48 and excluded those with positive screens < 24 h of admission, as those patients were thought to have symptoms of delirium prior to admission to the ICU. Secondary outcome measures included the type of early mobilization activities performed, perceived and identified barriers to early mobility, deferral and adverse events during rehabilitation interventions, and doses of sedative and analgesic infusions.

### Early Mobility Activities

At our institution, both OT and PT specialists are available to evaluate and treat ICU patients, but they require a formal and separate consult order to be placed by physicians or APs. PT services focus on gross motor skills such as transfers and ambulation whereas OT focuses on sensory stimulation, relaxation techniques, self-care training, and edema management. Both physical and occupational therapists provide therapies directed towards increasing strength and range of motion as well as functional mobility skills and splinting (**Supplementary Figure 2**).

An early mobility activity was defined as any activity intended to maintain or restore musculoskeletal strength and function that was performed within the first 72 h of admission to the ICU. Activities could be passive or active and included in-bed and out-of-bed interventions (**Box 1**) that were administered by rehabilitation therapists, nursing staff, or family members/caregivers. The level of activity was discussed during daily medical rounds, determined by the stability of a patient’s status and the amount of medical support required, and written on a communication board outside of the patient’s room (**Supplementary Figure 3**).

Box 1 **Types of mobilization activities provided by physical and occupational therapy, categorized into in-bed and out-of-bed activities.**

**Table:** 

IN BED ACTIVITIES	OUT OF BED ACTIVITIES
ROM (passive and active)	Use of mobility device
Bed positioning (passive and active)	Sit to stand
Splinting	Transfer (bed to chair/mat/caregiver’s arms)
Sitting at edge of bed	Pre-gait activities
Sensory stimulation	Ambulation
Relaxation techniques	Therapeutic play
Edema management	Self-care training
	Use of functional positioning device

### Statistical Analysis

A two-sample t-test was used in normally distributed data to test the difference in continuous variables by pre- and post- implementation periods. A Mann Whitney U test was used to test non-normally distributed continuous variables. A chi-square test was used to test the group difference for categorical variables. For categorical variables with relatively low frequencies in some subgroups (more than 30% cells have frequencies less than 5), Fisher’s exact test was used instead. A two-proportion z-test (two-tailed) was used to compare group differences in percentages, rates, and proportions. Analyses were conducted in R (R Core Team 2019, R Foundation for Statistical Computing) and SAS software, Version 9.4 (SAS Institute, Cary, NC).

## Results

### Demographics

The pre-implementation period included 294 patients, and the post-implementation period included 272 patients. The two groups had no significant differences in age or gender, and the median age was 8.7 (IQR 11.8) years. The pre-implementation group had significantly more surgical patients (24 vs 14%, *p*<0.05) and patients with underlying solid tumors (12 vs 8%, *p*<0.05) than did the post-implementation group, which had a significantly higher proportion of HCT patients (11 vs 23%, *p*<0.05). Median admission Pediatric Risk of Mortality (PRISM) scores in the post-implementation group [5.0 (IQR 8.0)] were significantly higher than those in the pre-implementation group [3.0 (IQR 8.0), *p*<0.05]. Other demographic and clinical variables were similar between the two groups (**Table 1**).

**Table 1:** Patient demographics during study period.

Patient Characteristic		Pre- implementation for all LOS (N=294)	Post- implementation for all LOS (N=272)	P value	Pre- implementation for LOS > 48 h (N=112)	Post-implementation for LOS > 48 h (N=120)	P value
Age in years, median (IQR)		8.35 (11.5)	10.0 (12.25)	0.709	6.4 (11.2)	9.05 (12.1)	0.308
Sex							
	Male	161	151	0.857	61	70	0.553
	Female	133	121		51	50	
Admission Type							
	Surgical/post-operative	71	38	0.002	18	13	0.241
Medical	HCT	32	62	0.001	16	30	0.064
	Leukemia/Lymphoma	103	88	0.063	39	37	0.313
	Solid Tumor	36	22	0.031	19	10	0.029
	Neuro-oncology	33	39	0.584	14	15	0.86
	Hematology	19	21	0.864	6	13	0.163
	Radiation oncology	0	2	0.499	0	2	0.5
ICU LOS, median (IQR)		2.0 (3.0)	2.0 (3.0)	0.614			
Admission PRISM score, median (IQ)		3.0 (8.0)	5.0 (8.0)	0.024			
Readmissions, N		3	4	0.631			
Ventilator days^a^, median (IQ)		3.0 (4.0) (N=52)	3.0 (5.0) (N=66)	0.983			

LOS, length of stay; SD, standard deviation; HCT, hematopoietic stem cell transplant; ICU, intensive care unit; PRISM, Pediatric Risk of Mortality. ^a^Ventilator days only reflect those patients who required mechanical ventilation.

### Primary Outcomes

For all admissions regardless of LOS, consults for rehabilitation services increased from 25% pre-BRAVE implementation to 56% (*p*<0.001) post-implementation. Additionally, the percentage of patients who received at least one mobility activity with PT and/or OT within 72 h of admission increased from 21 to 30% (*p*=0.02). In patients with LOS > 48 h, consults for rehabilitation services and percent of patients mobilized within 72 h increased from 34 to 67% (*p*<0.001) and from 29 to 35% (*p*=0.29), respectively. A positive delirium screen, defined as a CAPD score ≥ 9, was present in 43% of patients in the pre-implementation timeframe and 37% in the post-implementation timeframe (*p*=0.46; **Table 2**).

**Table 2:** Primary outcome measures.

Outcome Measure		Pre- implementation for all LOS (N=294)	Post- implementation for all LOS (N=272)	P value	Pre- implementation for LOS > 48 h (N=112)	Post-implementation for LOS > 48 h (N=120)	P value
Rehab Outcomes							
	Rehab consults placed within 72 h of admission	74	152	<0.001	38	80	<0.001
	Patients mobilized within 72 h of admission	62	81	0.017	32	42	0.294
Delirium Outcome							
	Positive delirium screen (CAPD≥9)				26/60	27/73	0.459

LOS, length of stay; CAPD, Cornell Assessment of Pediatric Delirium.

### Secondary Outcomes

We achieved a significant increase in the percentage of patients who received at least one out-of-bed activity with PT/OT within the first 72 h of ICU admission (16 to 29%, p<0.001) and saw a trend towards an increase in the percentage of patients who received at least one in-bed activity (10 to 15%, p=0.1). Reasons for deferral of interventions by rehabilitation staff included refusal of child, refusal of caregiver, scheduling conflict with a diagnostic study or procedure, limitation of staff, limitation of equipment, and staff concern about a patient’s clinical status. After BRAVE implementation, there was a significant increase in the number of activities deferred because of caregiver refusal, conflict with a diagnostic test or procedure, and staff concern about the patient’s clinical status. There was no significant change in the number of unplanned extubations (0 vs 0.005% of ventilator days, *p*=0.26) after implementation of BRAVE, and no events involving the unintentional removal of central venous catheters, injury to patient, or injury to staff during mobilization activities. When we compared the average daily infusion rate of dexmedetomidine, fentanyl, ketamine, midazolam, morphine, and propofol between the pre- and post- implementation periods, we found a significant decrease in the average infusion rates of morphine (0.13 vs 0.1 mg/kg/h, *p*<0.001) and propofol (5.15 vs 3.9 mg/kg/h, *p*<0.001) and an increase in dexmedetomidine use (0.69 vs 0.78 µg/kg/h, *p*<0.001) after BRAVE implementation. However, average infusion rates of fentanyl (3.63 vs 5.03 µg/kg/h, *p*<0.001), ketamine (0.53 vs 0.81 mg/kg/h, *p*<0.001), and midazolam (0.22 vs 0.44 mg/kg/h, *p*<0.001) significantly increased in the post-implementation period (**Table 3**).

**Table 3:** Secondary outcome measures.

Outcome Measure		Pre-implementation (N=294)	Post- implementation (N=272)	P value
Activity Type				
	At least one in-bed	30	40	0.103
	At least one out-of-bed	48	80	<0.001
Deferral Reason				
	Child refusal	10	17	0.118
	Caregiver refusal	1	7	0.0244
	Schedule conflict with a diagnostic test/procedure	4	23	<0.001
	Provider concern	13	41	<0.001
	Lack of staff	0	0	N/A
	Lack of equipment	2	1	0.6101
	Other (e.g. sleeping, agitated, restricted due to SARS-CoV2)	27	75	<0.001
Adverse Events				
	Unplanned extubation	0	2	0.2585
	Unintentional removal of CVC	0	0	N/A
	Staff injury	0	0	N/A
	Patient injury	0	0	N/A
Sedation (average daily infusion rate)				
	Dexmedetomidine (µg/kg/hr)	0.69	0.78	<0.001
	Fentanyl (µg/kg/hr)	3.63	5.03	<0.001
	Ketamine (mg/kg/hr)	0.53	0.81	<0.001
	Midazolam (mg/kg/hr)	0.22	0.44	<0.001
	Morphine (mg/kg/hr)	0.13	0.1	<0.001
	Propofol (mg/kg/hr)	5.15	3.9	<0.001

CVC, central venous catheter.

### Post-Implementation Staff Survey

Fifty-one staff members responded to the post-implementation survey. Of these, 20 were registered nurses, 7 respiratory therapists, 7 physical therapists, 6 advanced nurse practitioners, 4 physicians, 3 child life specialists, 2 occupational therapists, 1 speech therapist, and 1 patient care assistant. Twenty-five responders had worked in a critical care unit for >10 years. Forty-seven (92%) reported that the BRAVE early mobility initiative had had a positive impact on their patients and caregivers, and 33 (65%) identified a collaborative interprofessional approach as the most helpful aspect in mobilizing patients. Forty-three (84%) thought that the pediatric ICU moderately or fully supported the implementation of BRAVE and thirty-five (69%) felt that they were able to prioritize and actively incorporate mobility as part of their patients’ daily plan. The most cited barrier to mobility identified in the survey was a lack of resource and staffing (27.5%), followed by lack of support or prioritization (19.6%) and risk of unplanned extubations (17.6%).

## Discussion

In addition to the common risk factors for developing PICS-p, critically ill pediatric patients with underlying oncologic and hematologic disorders have additional risk factors, such as glucocorticoid-related immunosuppression, myopathy, and neurocognitive changes; high acuity of disease; chronic organ dysfunction; and high rates of critical care resource utilization (6, 7, 11, 22, 42–44). These factors highlight the need for interventions to recognize and address PICS-p in this vulnerable patient population. We used the ABCDEF ICU liberation bundle framework and an established pediatric ICU early mobility program (PICU Up)! to develop and implement an early mobility initiative that would optimize early rehabilitation in a pediatric onco-critical care unit. To our knowledge, we report the first use, safety, and feasibility of a multidisciplinary early mobility initiative in a pediatric onco-critical care unit.

The BRAVE early mobility initiative was designed with the global aim of decreasing the effects of PICS-p within a pediatric onco-critical care unit. Our SMART aims were to increase the number of patients mobilized within 72 h of ICU admission from 21 to 80%, and to decrease the percentage of patients with positive delirium scores after 24 h of ICU admission from 43 to 30% by 9 months post-implementation. Although we did not achieve our SMART aims during this time, we were able to significantly increase the consults placed for rehabilitation services and early mobilization of patients with all LOS and saw trends toward an increase in early mobilization in patients with LOS > 48 h and a decrease in positive delirium screens.

After multiple PDSA cycles to address the key drivers for our SMART aims, we identified three interventions that were central in moving closer to achieving these targets: standardizing PT/OT consult orders at the time of ICU admission, empowering nursing staff to lead the discussion of mobility and delirium at medical rounds, and tasking nursing staff to document delirium scores on all patients in the electronic medical records.

As with any new and innovative initiative, we encountered various intrinsic and extrinsic barriers associated with implementation of BRAVE, many of which were similar to those previously reported by others (45–49). Identified barriers to the implementation of BRAVE included a lack of resource and staffing, resistance to a change in ICU culture, and system-related processes.

The early mobility initiative appeared to be safe, as no serious adverse events were reported, and no patients experienced dislodgement or removal of a vascular access device during mobilization activities. Although two unintentional extubations occurred in the post-implementation period, a comprehensive review determined that neither were related to mobilization activities or inadequate sedation or resulted in clinical deterioration.

**Figure 4:**
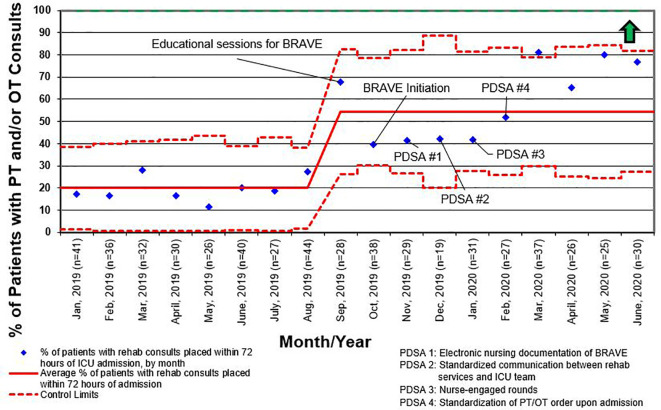
Control chart for the percentage of patients who had consults for physical therapy (PT) and/or occupational therapy (OT) placed within 72 h of admission to the intensive care unit (ICU) for all length of stay. BRAVE, Beginning Restorative Activities Very Early; PDSA, Plan-Do-Study-Act.

**Figure 5:**
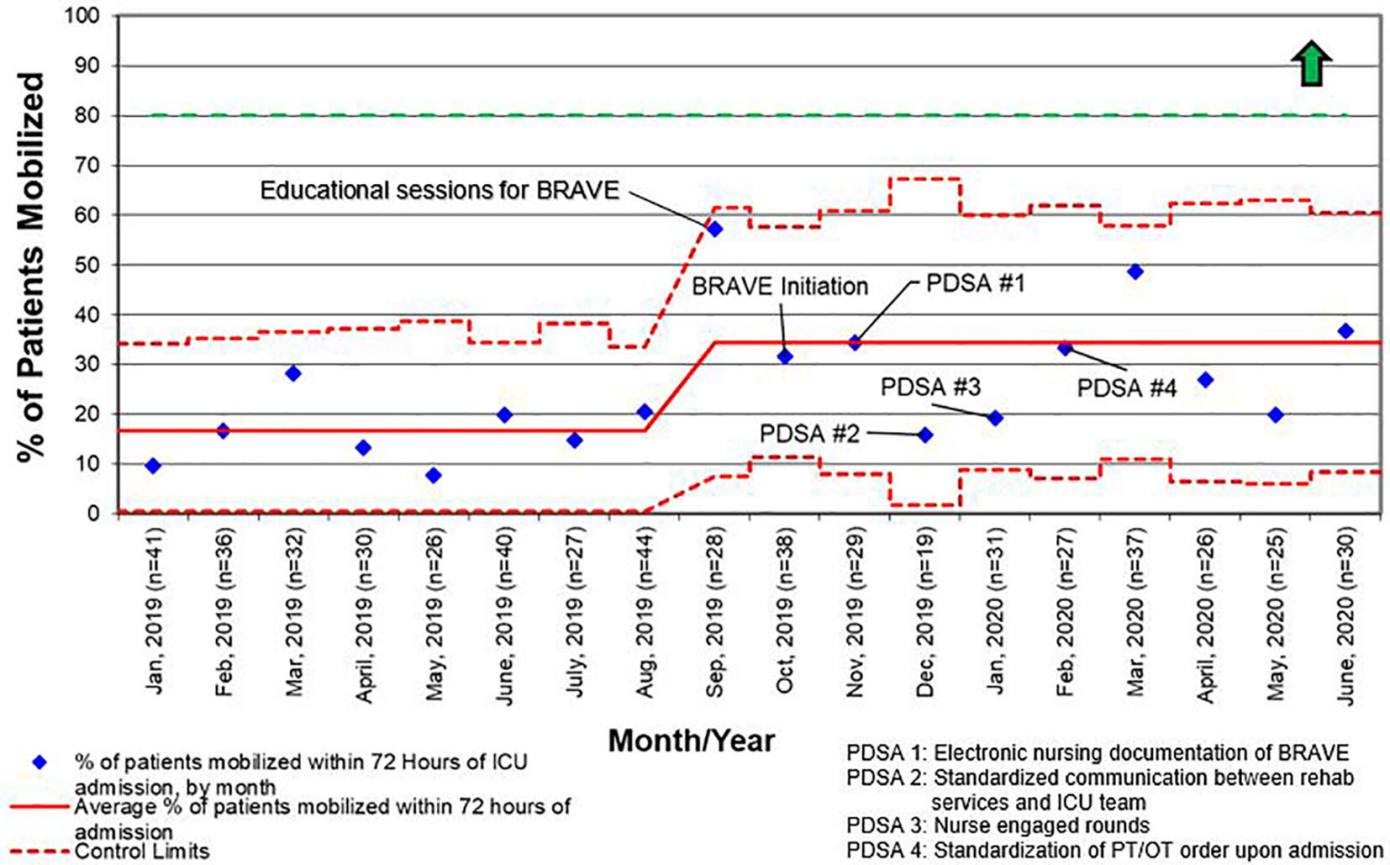
Control chart for the percentage of patients who were mobilized within 72 h of admission to the intensive care unit (ICU) for all length of stay. BRAVE, Beginning Restorative Activities Very Early; OT, occupational therapy; PT, physical therapy; PDSA, Plan-Do-Study-Act.

Like other children’s hospitals, our pediatric ICU does not have dedicated PT and OT providers (30). This limitation was magnified between March and May of 2020, when the SARS-CoV2 pandemic caused drastic reductions staffing of rehabilitation services. Although a lack of staff was not explicitly identified as a reason for deferring rehabilitation interventions during the study period, the significant increase in activity deferral from scheduling conflict with a diagnostic test or procedure in the post-implementation period emphasizes the need for dedicated ICU rehabilitation staffing who have flexibility in intervention timing. Additionally, a lack of resources and rehabilitation staff was identified as the most common barrier to early mobilization in the post-implementation survey. This deficit is illustrated by the differential increase in the percentage of patients with early rehabilitation consults placed when compared to the percentage of patients who were mobilized early (**Figures 4** and **5**). To address this shortfall, we are implementing a two-tiered approach for continued rehabilitation involvement after an initial assessment. The initial rehabilitation assessment occurs either *via* telehealth or in person and is followed by continued in-person therapy when skilled intervention is required. Otherwise, the nurse and families are provided with written instructions for mobility activities. As a part of this initiative, nurses, other staff members, and caregivers were educated and empowered to actively participate in mobility activities, an essential aspect of early mobility in critically ill children (28, 29).

Hesitancy to change in ICU culture was one of the largest barriers we encountered in this process. It proved essential to establish an interprofessional, collaborative group early in the development stage of the initiative. Thus, arising concerns could promptly be addressed on both an individual and departmental level. The multidisciplinary input allowed us to create small but lasting changes in culture and practice through serial PDSA cycles that targeted an 8-stage model of change (21, 50, 51). Recognition and celebration of staff efforts and positive outcomes through media outreach and engagement of hospital leadership were an integral part of generating widespread buy-in and maintaining patient and staff morale; however, ongoing education for all involved remains crucial to sustaining this initiative.

**Figure 6:**
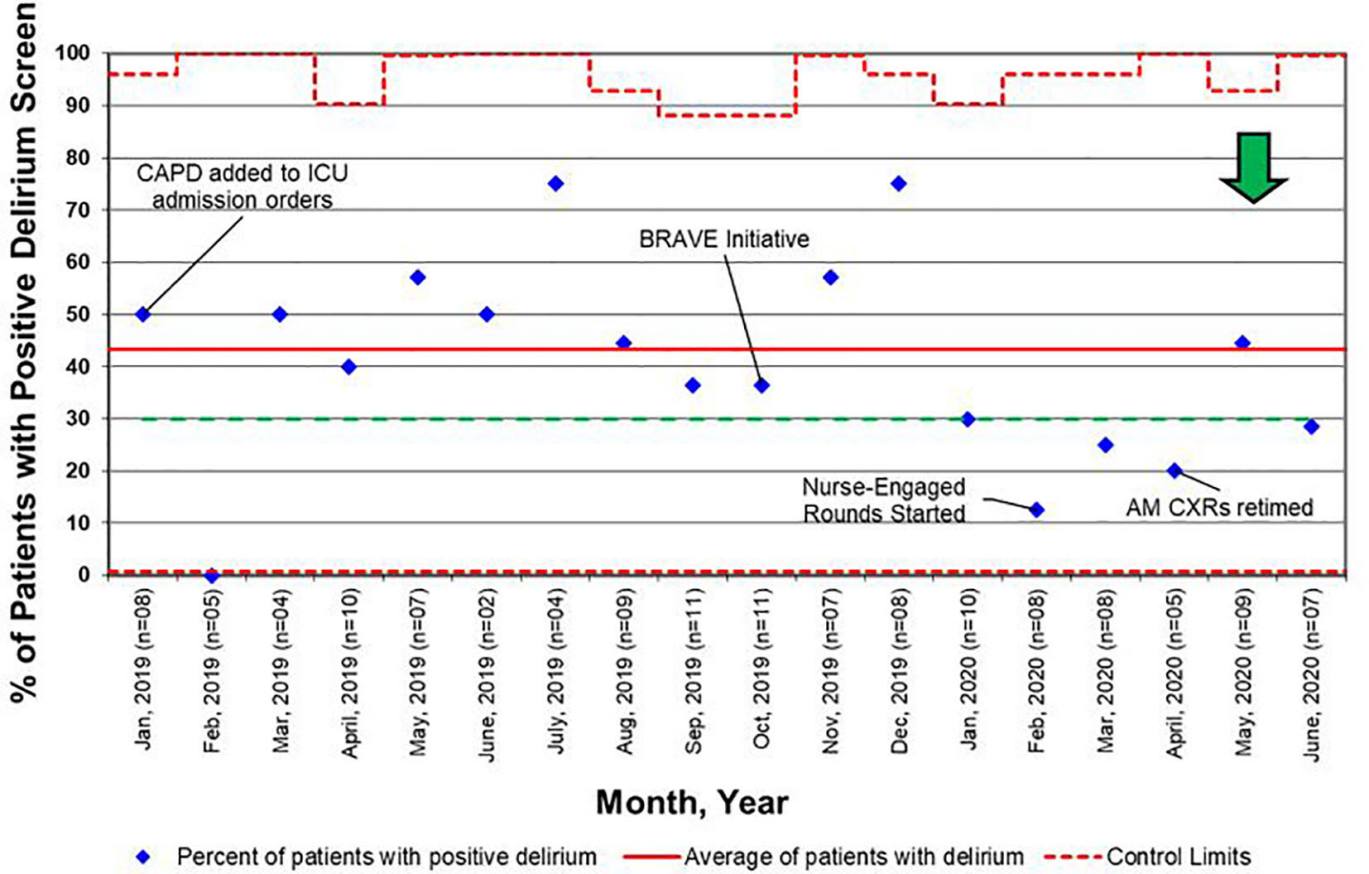
Control chart for delirium screens, as defined as a Cornell Assessment of Pediatric Delirium (CAPD) score ≥ 9. BRAVE, Beginning Restorative Activities Very Early; CXRs, chest x-rays; ICU, intensive care unit.

Another barrier we faced in providing patients with early mobility activities was related to system-based processes such as order entry and documentation. Initially, consult orders for PT and OT were placed independently at the discretion of physicians and APs. We carried out a PDSA cycle to standardize consult order entry for rehabilitation services as part of the initial admission orders. Additionally, by tasking nurses to assess, document, and discuss CAPD scores on all patients, delirium was recognized and addressed earlier, contributing to the decrease in overall positive delirium scores (**Figure 6**).

An unexpected finding in the post-implementation period of BRAVE pertains to use of opiate and sedative infusions. Although dosages of morphine and propofol decreased after implementation, fentanyl and midazolam dosages showed trends towards increasing during this period. The increase in fentanyl and midazolam use may be reflective of the differences in patient characteristic between the pre- and post- cohorts, specifically the significantly higher admission PRISM scores and proportion of HCT patients in the post-implementation period. However, to contend with this increase, we are implementing a nursing-led, Richmond Agitation Sedation Scale (RASS)-based sedation protocol (52, 53). As this PDSA cycle is ongoing, additional investigation is needed to examine the impact of BRAVE, particularly standardization of sedation scoring, on opiate analgesic and sedation usage.

Our study was limited by several factors. Because this was single- center study in an onco-critical care unit, our results may not be generalizable to a wider patient population. We reviewed data from 9 months pre- and post- BRAVE implementation in an 8-bed unit, thereby limiting the number of patients in this analysis. Also, data regarding mobilization activities was obtained retrospectively and relied on previous documentation. We also did not evaluate the effect of the increased workload and resource utilization that this initiative required, which is an important factor in interpreting the results of this study. Further, given this study’s time period coincided with our institution’s response to the global SARS-CoV2 pandemic, factors related to our hospital’s policy and staffing changes may have affected this data.

## Conclusion

Critically ill children with underlying oncologic and hematologic disorders form a unique population at high risk of developing PICS-P. Through a multidisciplinary team approach to ICU liberation in these patients, we have effectively and safely implemented an early mobility initiative in our onco-critical care unit. While our efforts support the safety and feasibility reported for such initiatives in other pediatric centers, more research is required to evaluate the effects of early mobility on patient outcomes, such as patient mortality, ICU LOS, ventilator-free days, incidence of pressure ulcers, and long-term neurocognitive and functional outcomes. Ongoing and future efforts include continuing to improve patient, family, and provider education, standardizing objective sedation scoring, and empowering caregivers and other providers to participate in patient mobility.

## Data Availability Statement

The raw data supporting the conclusions of this article will be made available by the authors, without undue reservation.

## Ethics Statement

Written informed consent was obtained from the minor(s)’ legal guardian/next of kin for the publication of any potentially identifiable images or data included in this article.

## Author Contributions

SGh, SW, AB, SB, KP, AC, LP, SS, KC, AE, MF, JR, MLe, and SK contributed to the conceptualization of the initiative and study design. SGh, KF, SW, AB, SB, AC, LP, SS, MJ, JS, KC, AE, MF, DT, JB-S, CB, MLo, JM, MD, MM, LH, and JR contributed to the development of PDSA cycles and implementation of the initiative. RM provided administrative support for the initiative. SGh, SW, AB, KP, SGo, RQ, AC, LP, SS, MLo, LH, and YA contributed to data collection. SGh, KF, SW, AB, KP, SGo, RQ, FW, HP, and MLe contributed to data analysis. SGh, KF, AB, FW, and HP contributed to writing the original draft of the manuscript. SGh, KF, SW, KP, and MLe contributed to making the figures and tables. SW, AB, KP, SGo, RQ, AC, LP, SS, SB, MJ, JS, KC, AE, MF, DT, JB-S, CB, MLo, JM, MD, MM, LH, JR, YA, JB, MLe, RM, and SK provided critical revisions to the manuscript. All authors contributed to the article and approved the submitted version.

## Conflict of Interest

The authors declare that the research was conducted in the absence of any commercial or financial relationships that could be construed as a potential conflict of interest.
