# Supplementary figures and images for: Beginning Restorative Activities Very Early: Implementation of an Early Mobility Initiative in a Pediatric Onco-Critical Care Unit
Source: Front Oncol. 2021 Mar 8;11:645716. doi: 10.3389/fonc.2021.645716 (PMC7982584; doi:10.3389/fonc.2021.645716)

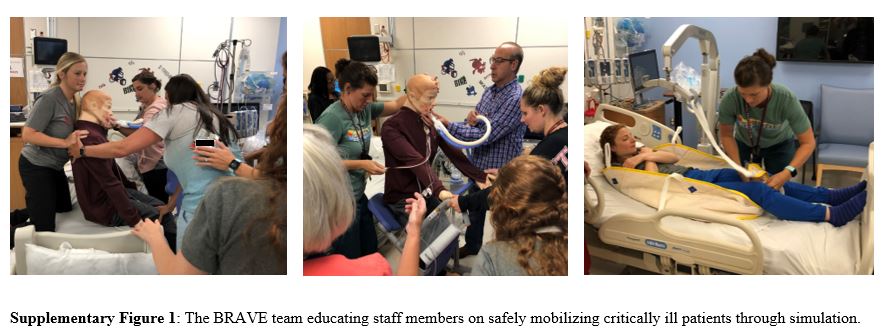

Supplement: Supplementary file 1 [file Image_1.jpeg]

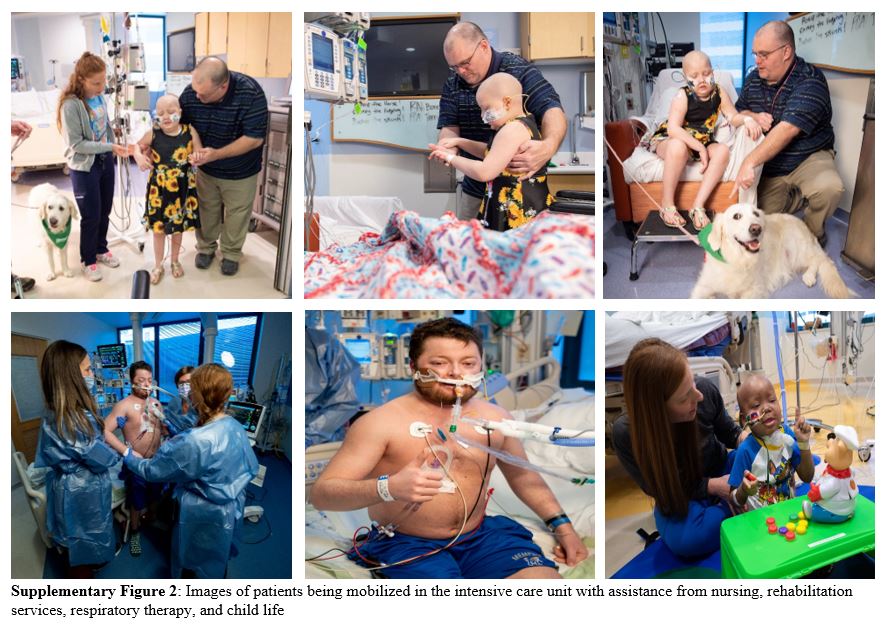

Supplement: Supplementary file 2 [file Image_2.jpeg]

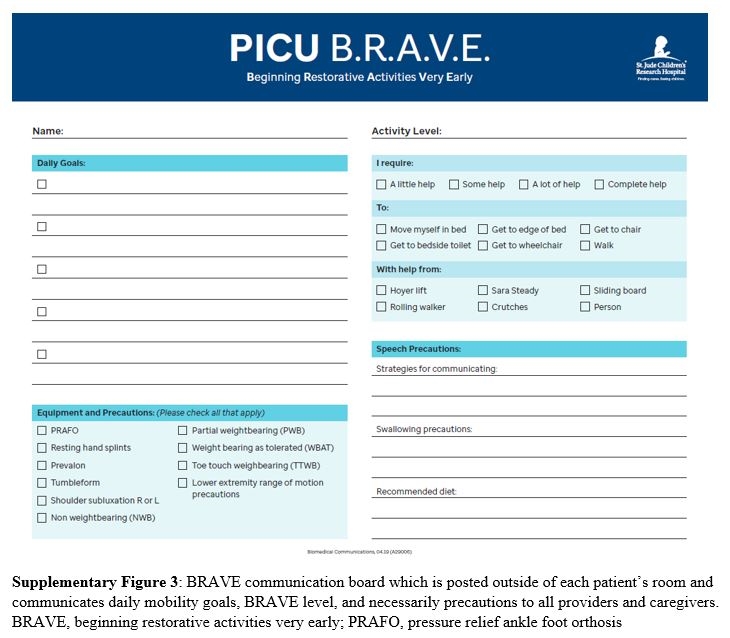

Supplement: Supplementary file 3 [file Image_3.jpeg]
